# Telmisartan Induces Growth Inhibition, DNA Double-Strand Breaks and Apoptosis in Human Endometrial Cancer Cells

**DOI:** 10.1371/journal.pone.0093050

**Published:** 2014-03-25

**Authors:** Naoko Koyama, Yoshihiro Nishida, Terukazu Ishii, Toshie Yoshida, Yuichi Furukawa, Hisashi Narahara

**Affiliations:** Department of Obstetrics and Gynecology, Oita University Faculty of Medicine, Oita, Japan; University of Quebec at Trois-Rivieres, Canada

## Abstract

Telmisartan, an angiotensin II receptor type 1 blocker, is often used as an antihypertension drug, and it has also been characterized as a peroxisome proliferator-activated receptor-gamma (PPARγ) ligand. The purpose of this study was to elucidate the antitumor effects of telmisartan on endometrial cancer cells. We treated three endometrial cancer cell lines with various concentrations of telmisartan, and we investigated the effects of the telmisartan on the cell proliferation, apoptosis, and their related measurements in vitro. We also administered telmisartan to nude mice with experimental tumors to determine its in vivo effects and toxicity. All three endometrial cancer cell lines were sensitive to the growth-inhibitory effect of telmisartan. The induction of apoptosis was confirmed in concert with the altered expression of genes and proteins related to the apoptosis. We also observed that DNA double-strand breaks (DSBs) were induced in HHUA (human endometrial cancer) cells by telmisartan treatment. In addition, experiments in nude mice showed that telmisartan significantly inhibited human endometrial tumor growth, without toxic side effects. Our results suggest that telmisartan might be a new therapeutic option for the treatment of endometrial cancers.

## Introduction

Endometrial cancers are the most common malignant tumors of the female genital tract, and their incidence has increased in recent years [1], [2]. However, the search for agents effective in the treatment of advanced and recurrent endometrial cancers has been disappointing [2], [3]. Innovative approaches are thus needed for the treatment of endometrial cancer.

The nuclear hormone receptor peroxisome proliferator-activated receptor-gamma (PPARγ) and its ligands induce apoptosis in several types of cancers, including endometrial cancer [4]–[6]. Telmisartan is an angiotensin II receptor type 1 (AT1R) blocker (ARB) that is widely used as an antihypertensive drug. Benson et al. reported a structural resemblance between telmisartan and pioglitazone, a PPARγ ligand [7]. Telmisartan functions as a partial agonist of PPARγ ligand, activating the receptor to 25%–30% of the maximum level achieved by the full agonists pioglitazone and resiglitazone, in which telmisartan acts independently via AT1R interaction [7]. Telmisartan has been reported to have antiproliferative activity in prostate cancer and renal cell carcinoma [8]–[10]. The effect of telmisartan on gynecologic cancer cells has not yet been investigated.

The present study was designed to reveal, for the first time, the biologic and therapeutic effects of telmisartan on endometrial cancer. We examined whether this compound could mediate the inhibition of cell proliferation and the induction of apoptosis in endometrial cancer cell lines. We investigated whether DNA double-strand breaks (DSBs) are induced in HHUA (human endometrial cancer) cells by telmisartan treatment. We also tested the ability of telmisartan to inhibit the proliferation of HHUA cells in vivo, using a nude mouse model.

## Materials and Methods

### Cell Lines

The HHUA human endometrial cancer cell line was obtained from Riken (Ibaraki, Japan). The Ishikawa human endometrial cancer cell line was kindly provided by Dr. Masato Nishida (Tsukuba University, Ibaraki, Japan)[11]–[13]. The HEC-59 human endometrial cancer cell line and normal human adult dermal fibroblast cell line were obtained from the American Type Culture Collection (Manassas, VA, USA). HHUA, Ishikawa, HEC-59 cells were maintained as monolayers at 37°C in 5% CO_2_/air in Dulbecco’s modified Eagle’s Medium (DMEM; Gibco, Rockville, MD). Normal human adult dermal fibroblast cells were maintained as monolayers in minimum essential media (MEM; invitrogen, Carlsbad, CA ) at same condition described above. All cells were supplemented with 10% heat-inactivated fetal bovine serum (FBS; Omega, Tarzana, CA).

### Chemicals

Telmisartan, candesartan, losartan and valsartan were obtained from LKT Laboratories (St. Paul, MN, USA), and prepared as 10 mg/mL stock solutions in dimethyl sulfoxide (DMSO). GW9662 and troglitazone were obtained from Cayman Chemical (Ann Arbor, MI, USA). GW9662 was prepared as a 5 mg/mL stock solution in dimethyl formamide. Troglitazone was prepared as a 10 mg/mL stock solution in DMSO. The stock solutions were stored as aliquots at −20°C.

### Assessment of Cell Proliferation and Cell Viability

Cell proliferation and viability were determined in 96-well plates by a modified methylthiazol tetrazolium (MTT) assay using WST-1 (Roche Diagnostics, Penzberg, Germany) following the manufacturer’s protocol. We seeded 5×10^3^ cells in DMEM supplemented with 10% FBS into each well of a 96-well flat-bottomed microplate (Corning, New York, NY) and incubated them overnight. The medium was then removed, and the cells were incubated for 48 h with 100 μL of experimental medium containing various concentrations of telmisartan, candesartan, losartan and valsartan. Thereafter, 10 μL of WST-1 dye was added to each well, and the cells were further incubated for 2 h. All experiments were performed in the presence of 10% FBS. Cell proliferation was evaluated by measuring the absorbance at 450 nm. The reference wavelength was 620 nm. Data were calculated as the ratio of the values obtained for the telmisartan-treated cells to those for the untreated controls.

### Anticancer Effects of Telmisartan via the PPARγ-dependent Pathway

Next, we examined the function of antitumor effects of telmisartan through PPARγ in HHUA endometrial cancer cells in vitro, using a WST-1 assay with 2-day exposure to both telmisartan at 10 or 50 μM and a PPARγ antagonist, GW9662, at 10 μM. GW9662 was used as described previously [14], [15]. Before starting stimulation, we seeded 5×10^3^ HHUA cells in DMEM supplemented with 10% FBS into each well of a 96-well flat-bottomed microplate (Corning, New York, NY) and incubated them overnight. HHUA cells were pretreated with GW9662 for 30 min before stimulation of telmisartan.

### Measurement of Apoptosis (Flow-cytometric Analysis with the Annexin V/Propidium Iodide Assay)

Cells were plated and grown overnight until they reached 80% confluence and then treated with telmisartan. After 48 h, detached cells in the medium were collected, and the remaining adherent cells were harvested by trypsinization. The cells (1×10^5^) were washed with PBS and resuspended in 500 μL of binding buffer (annexin V-fluorescein isothiocyanate [FITC] apoptosis detection kit from BioVision, Palo Alto, CA) containing 5 μL of 50 μg/mL propidium iodide (PI) and 5 μL of annexin V-FITC, which binds to phosphatidylserine translocated to the exterior of the cell membrane early in apoptosis as well as during late in apoptosis. After incubation for 10 min at room temperature in a light-protected area, the samples were analyzed on a FACSCalibur flow cytometer (Becton Dickinson, Lincoln Park, NJ). FITC and PI emissions were detected in the FL-1 and FL-2 channels, respectively. For each sample, data from 10,000 cells were recorded in list mode on logarithmic scales. Subsequent analysis was performed with CellQuest software (Becton Dickinson).

### Mitochondrial Transmembrane Potential

Cells were prepared for FACS as described above and stained using a Mitocapture Apoptosis Detection kit from BioVision with a fluorescent lipophilic cationic reagent that assesses mitochondrial membrane permeability, according to the manufacturer’s recommendations.

### Caspase-3 and -7 Activities

Caspase-3 and -7 are major members of the cysteine aspartic acid-specific protease (caspase) family that play key effector roles in apoptosis in mammalian cells. The activities of these caspases were measured using a Caspase-Glo 3/7 Assay purchased from Promega (Madison, WI), according to the manufacturer’s recommendations.

### Enzyme-linked Immunosorbent Assay

Activated cleaved poly-ADP ribose polymerase (PARP) expression was calculated using a Pierce Colorimetric In-Cell ELISA kit (Thermoscientific, Rockford, IL), according to the manufacturer’s recommendations.

### Western Blot Analysis

Cells were washed twice in PBS and lysed using M-PER mammalian protein extraction reagent (Thermoscientific) according to the manufacturer’s recommendations. Protein concentrations were quantified using the Pierce 660-nm protein assay reagent (Thermoscientific) according to the manufacturer’s suggested protocol. Whole-cell lysates (20 μg) were resolved by SDS-polyacrylamide gel electrophoresis on 10%–15% gels, and transferred to polyvinylidene difluoride membranes (Immobilon; Amersham, Arlington Heights, IL). Membranes were then probed sequentially with antibodies against Bcl-2 (1∶1,000; Epitomics, Burlingame, CA), Bcl-xL (1∶1,000; Cell Signaling Technology), Bax (1∶1,000; Cell Signaling Technology), cleaved caspase-3 (1∶1000; Cell Signaling Technology), and GAPDH (1∶10,000; Abcam, Cambridge, UK). The blots were developed using an enhanced chemiluminescence substrate (Western Lightning ECL Pro) kit (PerkinElmer, San Jose, CA).

### Detection of DSBs by Pulsed-field Gel Electrophoresis (PFGE)

Subconfluent cultures of HHUA cells were treated with 10 or 100 μM telmisartan for 24 h or 48 h. Cells were harvested after trypsinization, and agarose plugs containing 10^6^ cells were prepared with a CHEF disposable plug mold (Bio-Rad, Hercules, CA). We incubated the plugs in lysis buffer (100 mM EDTA, 1% (w/v) sodium lauryl sarcosyne, 0.2% (w/v) sodium deoxycholate, 1 mg mL^−1^ proteinase K) at 37°C for 24 h and then washed them with 10 mM Tris-HCL, pH 8.0, 100 mM ETDA. Electrophoresis was performed for 23 h at 13°C through 0.9% agarose in Tris-borate-EDTA buffer using a Biometra Rotaphor apparatus (Biometra, Goettingen, Germany) with the following parameters: interval, 30–5 s log; angle, 120°–110° linear; 180–120 V log). The DNA was stained with ethidium bromide and visualized using a Typhoon FLA 7000 scanner (GE Healthcare Life Sciences, Tokyo, JAPAN). The electrophoresis conditions were specifically designed to compact lower-molecular-weight DNA fragments (several Mbp to 500 kbp) into a single band, while keeping high-molecular-weight genomic DNA in the well. The lower-molecular-weight DNA fragments are the results of DSBs in the chromosomal DNA. Thus, the assay allows broken DNA to be readily detected. However, in the context of DSBs arising during DNA replication stalling, the assay has limited sensitivity and is not quantitative. When a DSB occurs at a DNA replication fork, the broken DNA is still attached to the template chromosome. To detect DSBs in the assay, two relatively closely (several Mbp) spaced independent DSBs have to occur [16], [17].

### Immunofluorescent Staining of γ-H2AX

HHUA cells were treated with or without telmisartan fixed in 3.7% paraformaldehyde for 15 min, and then blocked with 0.5% BSA in PBS. The cells were incubated with rabbit monoclonal anti- γ-H2AX antibody (1∶500; Millipore, Billerica, MA) for 90 min at room temperature, followed by Alexa488-conjugated anti-rabbit IgG (1∶300; Molecular Probes, Carlsbad, CA) for 60 min at room temperature. DAPI was used for nucleus staining. The cells were observed using a BZ-9000 (Keyence, Osaka, Japan).

### *In vivo* Animal Treatment Protocol

The protocol was approved by the Committee on the Ethics of Animal Experiments of the University of Oita (Permit Number: L029001). All surgery was performed under sodium pentobarbital anesthesia, and all efforts were made to minimize suffering. All animal experiments were performed in compliance with National Institutes of Health guidelines.

Sixteen 6-week-old immunodeficient BALB/c-nu/nu female mice were purchased from Charles River Laboratories Japan (Yokohama, Japan) and maintained under pathogen-free conditions with irradiated chow. In one experiment, 5×10^6^ HHUA cells in 0.1 mL Matrigel (Collaborative Biomedical Products, Bedford, MA) were bilaterally and subcutaneously injected into the trunks of 16 mice, leading to the formation of two tumors per animal. Treatment started on the day after the injection of these human endometrial carcinoma cells and was discontinued after 7 weeks [18]. Cohorts (eight mice per group) received either diluent only (control group) or telmisartan (100 μg per day) intraperitoneally for 5 days per week. The tumors were measured every week with vernier calipers. We calculated the tumor volumes using the following formula: volume = length×width×height×0.5236. Left ventricle (LV) pressure was monitored using a pressure transducer (WS-681G: Nihon Kohden, Tokyo) to record the peak positive and negative first derivatives of LV pressure. Data were analyzed using LabChart software (ADInstruments, Colorado Springs, CO). Animals were sacrificed by sodium pentobarbital anesthesia, after which careful resection was performed and tumor weights were measured. Tumors were fixed and stained for histological analysis.

### Immunohistochemistry

Tumor specimens were fixed in 10% neutral buffered formalin and embedded in paraffin before histological sectioning. Immunohistochemistry studies were performed on formalin-fixed sections of tissue specimens. Sections were pretreated with trypsin (10 mg per 50 mL in Tris buffer, pH 8.1) for 10 min at 37°C, followed by incubation with anti-Ki-67 monoclonal antibody (1∶1000 dilution in PBS; Zymed Laboratories, San Francisco, CA) for 30 min. Slides were then washed in PBS and incubated sequentially for 15 min with peroxidase-conjugated swine anti-mouse immunoglobulin G (1∶50 dilution; Dako, Copenhagen, Denmark). Staining was performed using a Dako autostainer. Localization of reaction products was performed using the diaminobenzidene reaction.

### Terminal Deoxynucleotidyltransferase-mediated Uridine Triphosphate End-labeling Analysis

DNA strand breaks were identified via a terminal deoxynucleotidyltransferase-mediated uridine triphosphate end-labeling (TUNEL) analysis, using an In Situ Cell Death Detection Kit (Roche) in accord with the manufacturer’s instructions.

### Statistical Analysis

All experiments were performed independently at least three times in triplicate per experimental point. All numerical data are expressed as means ± SD. Significance was determined by conducting a *t*-test with Bonferroni correction. A *P* value of <0.05 was accepted as significant.

## Results

### Effects of Telmisartan on the Proliferation and Viability of Endometrial Cancer Cell Lines *in vitro*

**Figure 1:**
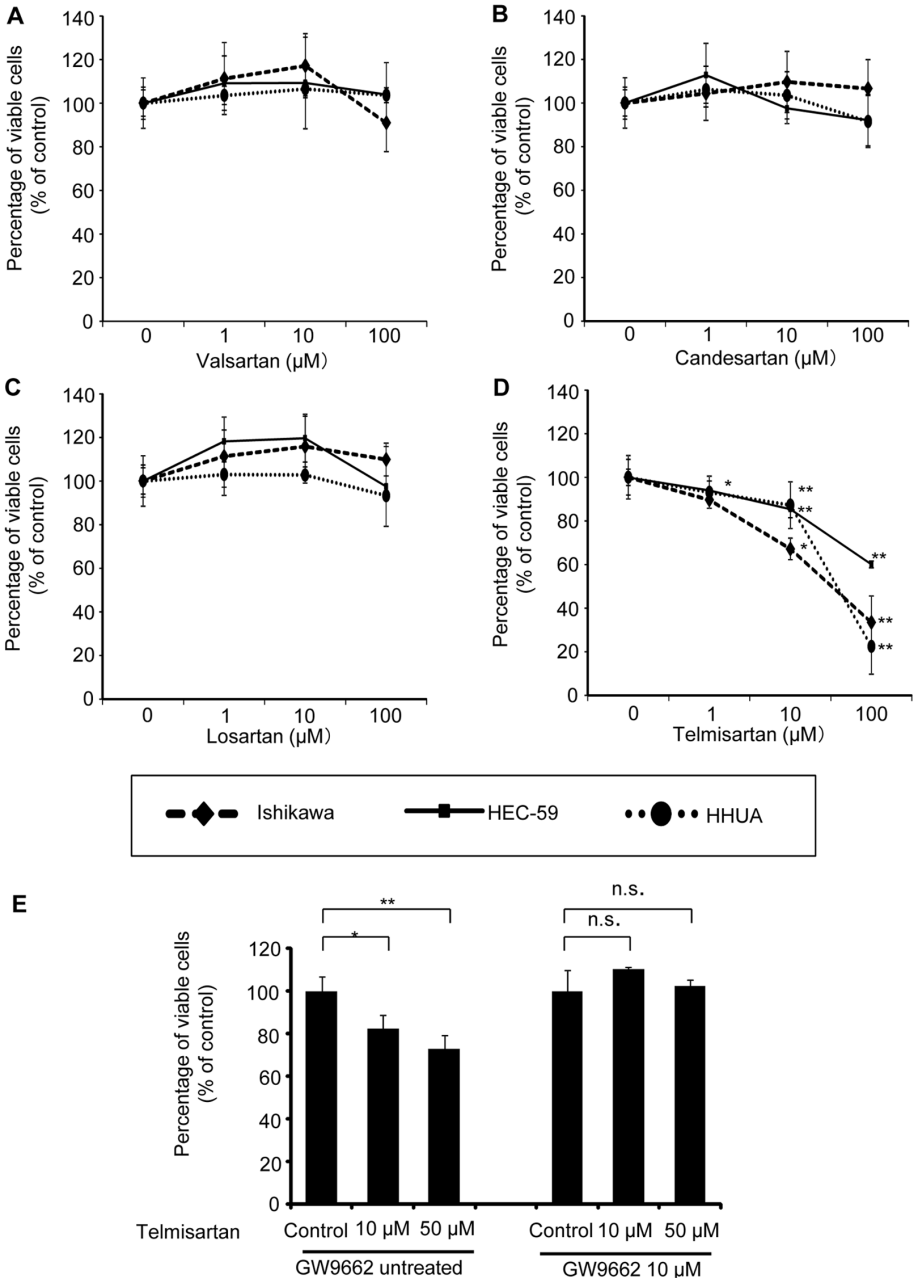
The effect of the ARBs telmisartan, valsartan, losartan and candesartan on the proliferation of endometrial cancer cell lines in vitro. Anticancer effects of telmisartan via the PPARγ-dependent pathway. (A–D) Ishikawa, HEC-59, and HHUA endometrial cancer cell lines were treated with telmisartan, candesartan, losartan or valsartan at various concentrations (1–100 μM) or the vehicle (control) for 48 h, and proliferation (% of control) was measured in a WST-1 assay. Results are means ± SD of three independent experiments with triplicate dishes. *P<0.05 vs. control, **P<0.01 vs. control. (E) Effect of GW9662 on telmisartan inhibition of HHUA cell proliferation. HHUA cells were incubated with telmisartan (10 or 50 μM) for 48 h followed by preincubation with GW9662 (10 μM) for 30 min. We found that the addition of GW9662 inhibited the anticancer effects of telmisartan at 10 or 50 μM. Results represent the means ± SD of three independent experiments. Columns, means; bars, SDs. *P<0.05 vs. control, **P<0.01 vs. control.

We examined the antitumor effects of telmisartan, candesartan, losartan and valsartan on three endometrial cancer cell lines in vitro, using a WST-1 assay with a 2-day exposure to these ARBs. Three endometrial cancer cell lines (Ishikawa, HEC-59, and HHUA) showed significant sensitivity to telmisartan treatment at 1–100 μM (Fig. 1D). None of the other three ARBs (candesartan, losartan and valsartan) affected the viability of the endometrial cancer cell lines (Fig. 1A–C). Since we found that telmisartan could inhibit the proliferation of these cell lines, we examined the function of the antitumor effects of telmisartan through PPARγ in HHUA endometrial cancer cells in vitro, using a WST-1 assay with 2-day exposure to both telmisartan at 10 or 50 μM and a PPARγ antagonist, GW9662, at 10 μM. We found that the addition of GW9662 inhibited the anticancer effects of telmisartan at 10 or 50 μM (Fig. 1E).

### Apoptotic Changes in the Endometrial Cancer Cells Treated with Telmisartan

To assess the ability of telmisartan to induce apoptosis in cancer cells and to help distinguish the different types of cell death, we double-stained telmisartan-treated cells with annexin V and PI and analyzed the results using flow cytometry. Annexin V binding combined with PI labeling was performed for the distinction of early apoptotic (annexin V+/PI−) and late apoptotic (annexin V+/PI+) cells [19]. After the treatment of endometrial cancer cells with telmisartan at 100 μM, we detected a simultaneous increase in both the annexin V+/PI− fraction (early apoptotic) and annexin V+/PI+ (late apoptotic) subpopulations (Table 1).

**Table 1:** Cell death measured by annexin V assay and mitochondrial transmembrane potential (MTP) assay in endometrial cancer cell lines.

	Cell Line		Vehicle	Telmisartan (100 μM)
Annexin V assay	Ishikawa	Viable (LL) (%)	87.1±2.9	60.4±0.6**
		Early Apoptosis (LR) (%)	2.2±1.5	3.1±1.1
		Late Apoptosis (UR) (%)	6.2±0.6	20.0±1.8**
	HEC-59	Viable (LL) (%)	87.2±6.8	42.1±9.6 **
		Early Apoptosis (LR) (%)	4.2±1.7	4.8±2.9
		Late Apoptosis (UR) (%)	3.4±2.1	14.7±2.7**
	HHUA	Viable (LL) (%)	92.9±1.8	43.1±7.6**
		Early Apoptosis (LR) (%)	2.9±0.6	6.6±2.0
		Late Apoptosis (UR) (%)	1.2±0.4	12.0±3.1*
MTP assay	Ishikawa	Viable (%)	86.9±4.9	70.7±5.1*
		Apoptosis (%)	13.5±5.1	30.1±5.3*
	HEC-59	Viable (%)	98.8±0.3	78.8±0.1**
		Apoptosis (%)	1.3±0.3	21.7±0.1**
	HHUA	Viable (%)	92.7±1.4	73.1±0.4**
		Apoptosis (%)	7.5±1.4	27.3±0.4**

Endometrial cancer cells were treated with 100 μM of telmisartan for 48 h. Cells were then stained with annexin V and PI (propidium iodide). The positive cells were detected by flow cytometry. The viable cells were negative for both annexin V and PI staining (the lower left quadrant of the cytograms, LL), the early apoptotic cells were positive for annexin V staining but negative for PI staining (the lower right quadrant, LR), and the late apoptotic cells were positive for both annexin V and PI staining (the upper right quadrant, UR). MTP results were analyzed using the MitoCapture assay. Endometrial cancer cells were treated with telmisartan for 48 h and harvested for flow cytometry. Each experiment was repeated three times. Results = means ± SD of three independent experiments. *P<0.05, **P<0.01 vs. the control group.

### Loss of Mitochondrial Transmembrane Potential in Response to Treatment with Telmisartan

Loss of mitochondrial transmembrane potential (MTP) has been shown to occur prior to nuclear condensation and caspase activation, and is linked to cytochrome c release in many but not all apoptotic cells [20]. Treatment of endometrial cancer cells with telmisartan resulted in a decrease in MTP (Table 1).

### Effects of Telmisartan on the Expression of Apoptosis-related Proteins

**Figure 2:**
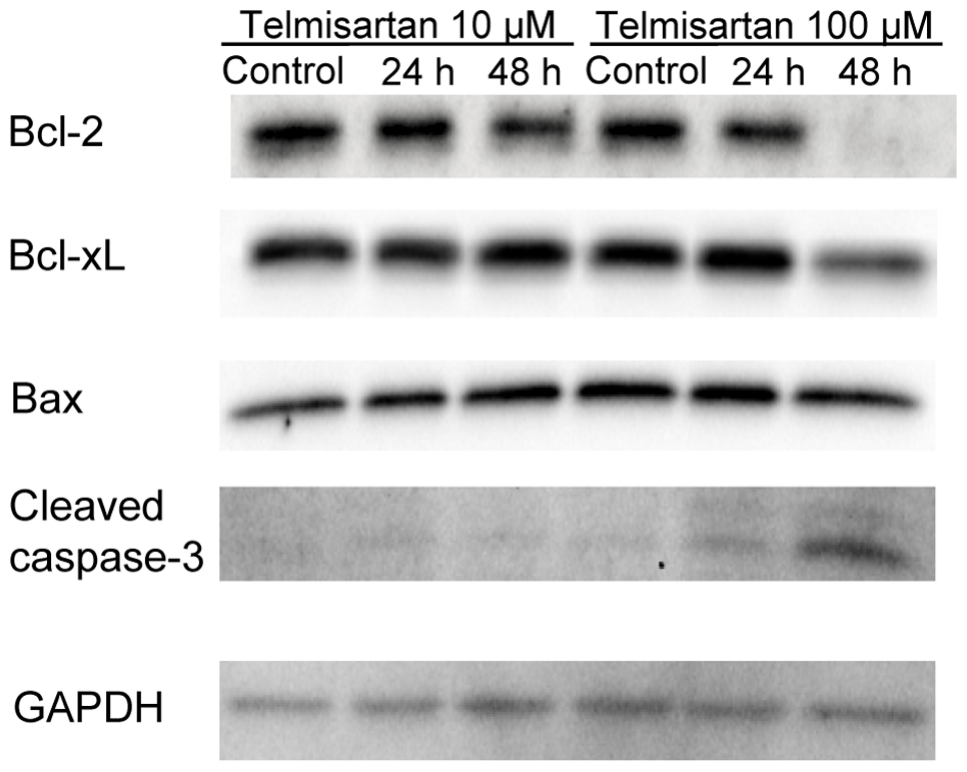
Expression of the apoptosis-related genes and proteins treated with telmisartan in endometrial cancer cells. HHUA cells were treated with telmisartan at 10 or 100 μM, and cell lysates were harvested after 24 and 48 h. A Western blot analysis was performed with a series of antibodies (Bcl-2, Bcl-xL, Bax, cleaved caspase-3 and GAPDH). Control cells were treated with vehicle alone.

We examined the effects of telmisartan on the expression of apoptosis-related proteins in endometrial cancer cell lines by a Western blot analysis (Fig. 2). The Bcl-2 family of apoptosis-regulating proteins functions to either promote (Bax, Bad, and Bak) or inhibit (Bcl-2, Bcl-xL, and Mcl-1) the apoptotic response to a wide variety of stimuli including chemotherapy and radiotherapy [21]. Telmisartan at 100 μM for 48 h decreased the expression of Bcl-2 and Bcl-xL, in which cleaved fragments of caspase-3 were detected (Fig. 2).

### Effects of Telmisartan on DNA Damage Response and Cell Death

**Figure 3:**
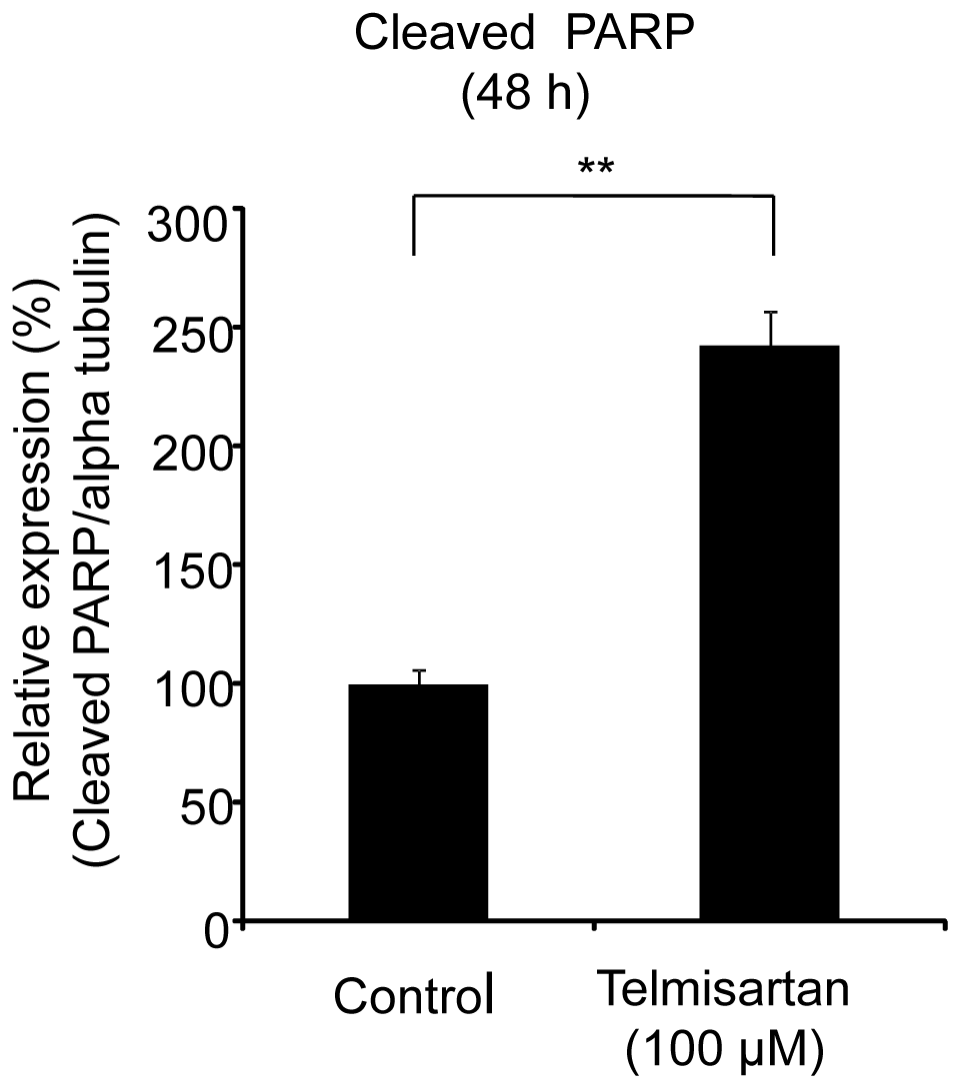
Expression of the cleaved PARP treated with telmisartan in endometrial cancer cells. The protein expression of cleaved PARP was measured by ELISAs. HHUA cells were treated with 100 μM telmisartan for 48 h. Control cells were treated with vehicle alone. Results represent the means ± SD of three independent experiments. Columns, means; bars, SDs. **P<0.01 vs. control.

**Figure 4:**
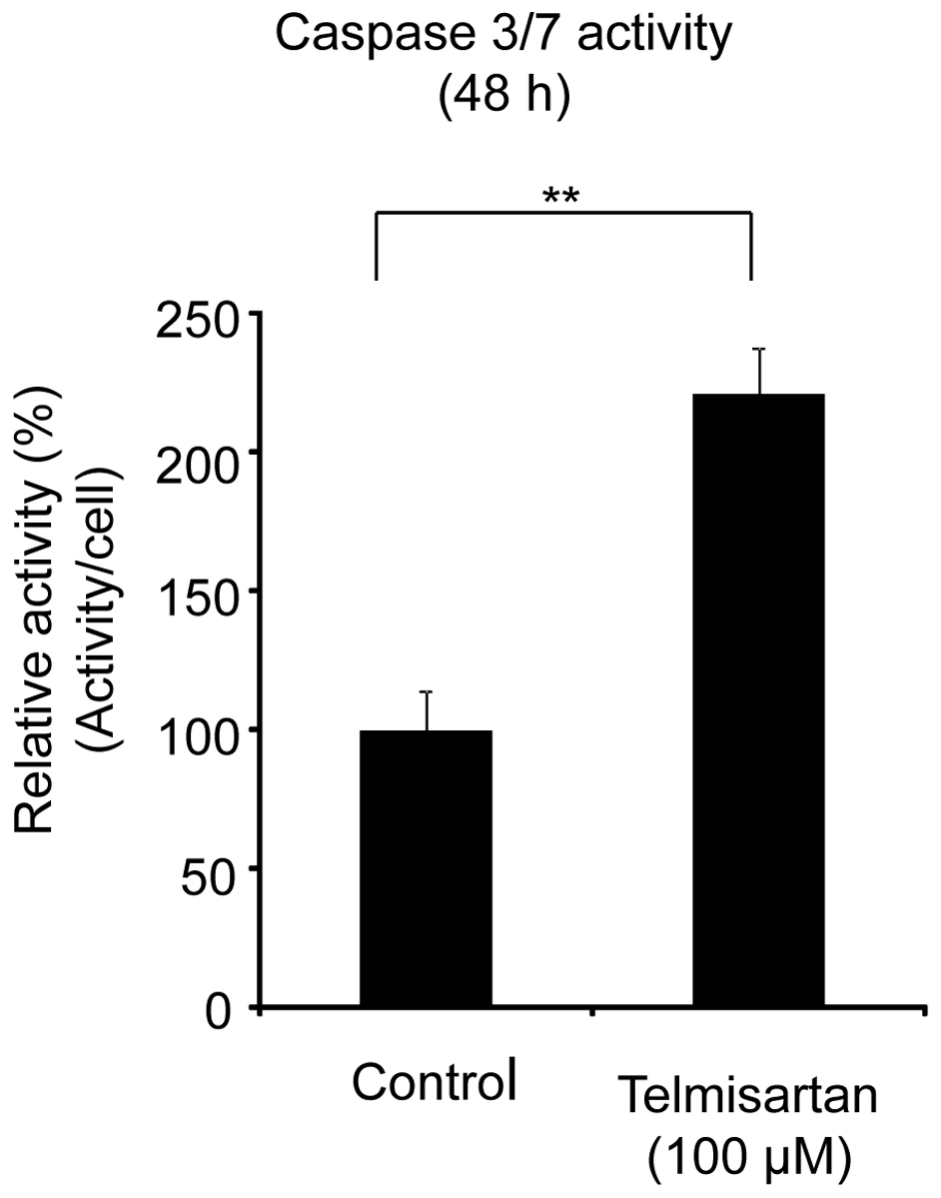
The activities of caspase-3 and caspase-7 treated with telmisartan in endometrial cancer cells. The activities of caspase-3 and caspase-7 were assessed by Caspase-Glo 3/7 Assays. HHUA cells were treated with 100 μM telmisartan for 48 h. Control cells were treated with vehicle alone. Results represent the means ± SD of three independent experiments. Columns, means; bars, SDs. **P<0.01 vs. control.

**Figure 5:**
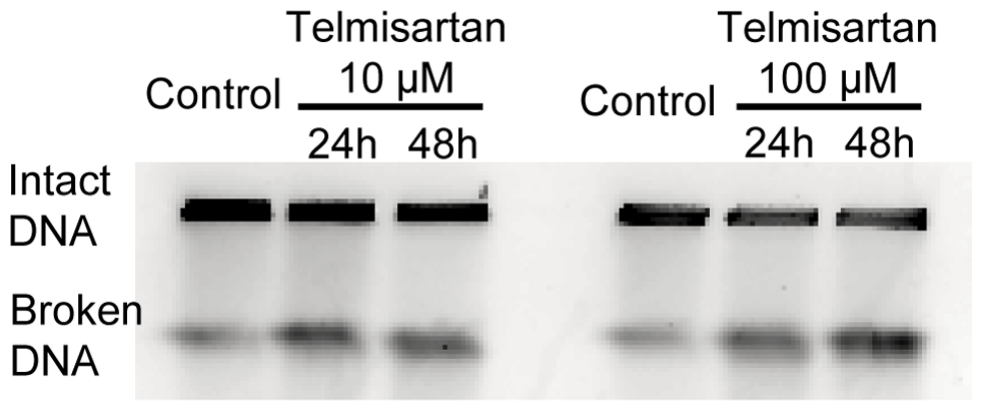
Analysis of telmisartan-induced DSB formation in HHUA cells. DSB formation was analyzed by PFGE. HHUA cells collected into agarose plugs, and their DNA was separated by size on an agarose gel. Under the electrophoresis conditions used, high-molecular-weight genomic DNA remained in the well, whereas lower-molecular-weight DNA fragments (several Mbp to 500 kbp) migrated into the gel and were compacted into a signal band.

**Figure 6:**
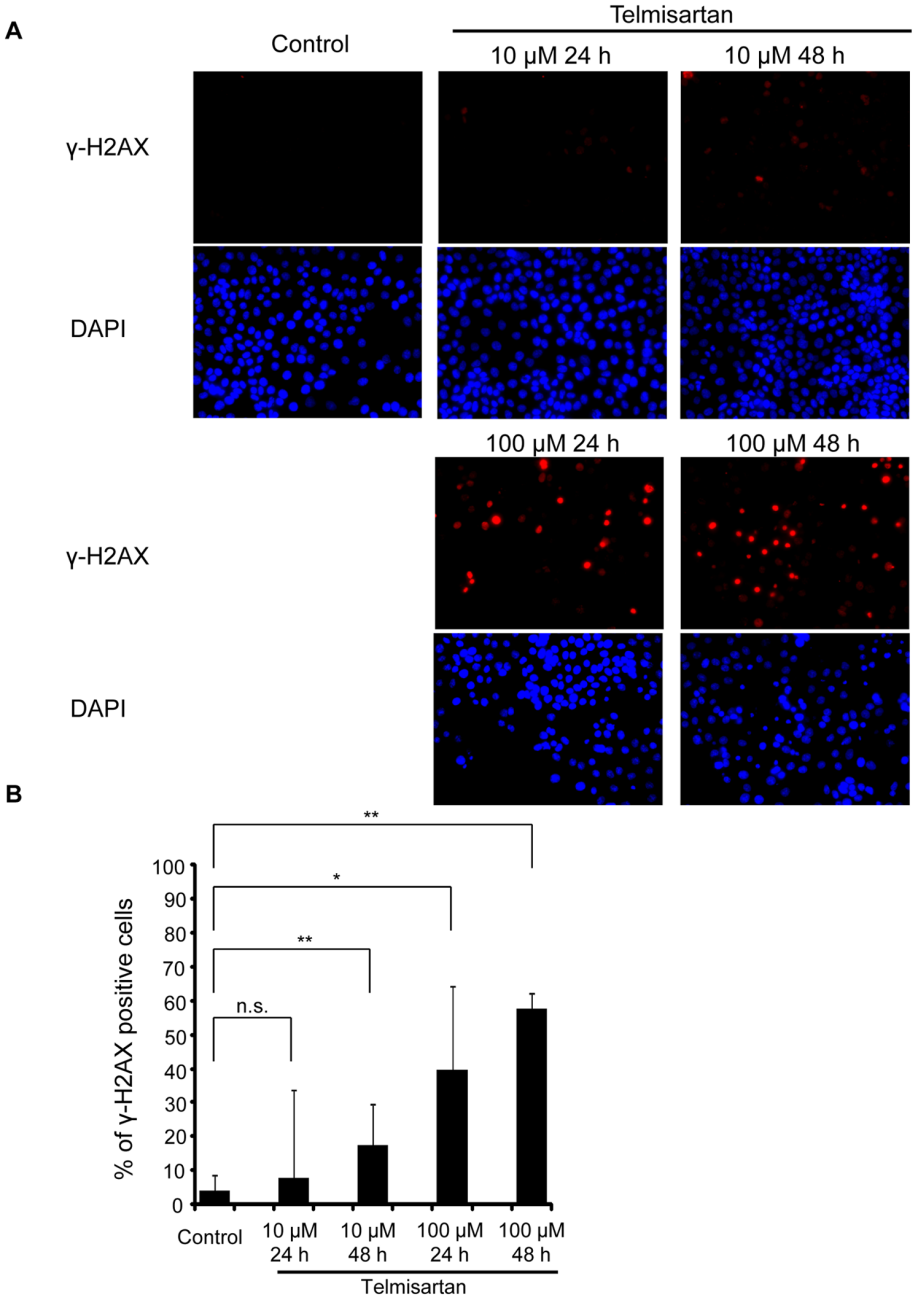
Immunofluorescent staining of γ-H2AX. (A) Time and dose dependency of histone H2AX phosphorylation by telmisartan. HHUA cells were treated with 10 or 100 μM telmisartan for indicated times followed by immunostaining using an anti-γ-H2AX antibody. Their nuclei were revealed by DAPI staining. (B) Percentage of γ-H2AX-positive cells. HHUA cells were treated with 10 or 100 μM telmisartan for 24 h or 48 h. Control cells were treated with vehicle alone. The telmisartan-treated cells had significantly higher levels of fluorescence compared to the untreated cells. Results = means ± SD of three independent experiments. Columns, means; bars, SDs. *P<0.05 vs. control, **P<0.01 vs. control.

The expressions of cleaved PARP were examined by ELISA after the treatment of HHUA cells with telmisartan for 48 h. Telmisartan (100 μM) markedly increased the levels of cleaved PARP in HHUA cells (Fig. 3). To elucidate the activity of effectors in telmisartan-induced apoptosis, we used a Caspase-Glo 3/7 Assay, which measures caspase-3 and -7 activities. After the treatment of HHUA cells with telmisartan at 100 μM for 48 h, caspase-3 and -7 activities were upregulated (Fig. 4). We tested telmisartan-induced DSB formation in HHUA cells. HHUA cells were treated with 10–100 μM telmisartan for 24 or 48 h, and DSBs were detected using pulsed-field gel electrophoresis (PFGE). After the telmisartan treatment, broken DNA was increased in a dose-dependent manner in HHUA cells (Fig. 5). Next, we performed immunofluorescent staining of γ-H2AX to detect DSBs. The telmisartan-treated HHUA cells had significantly higher levels of fluorescence compared to the untreated cells. As shown in Figure 6A, B, γ-H2AX was upregulated in the HHUA cells after incubation with telmisartan at 10–100 μM for 24 or 48 h.

### Antitumor Activity *in vivo*

**Figure 7:**
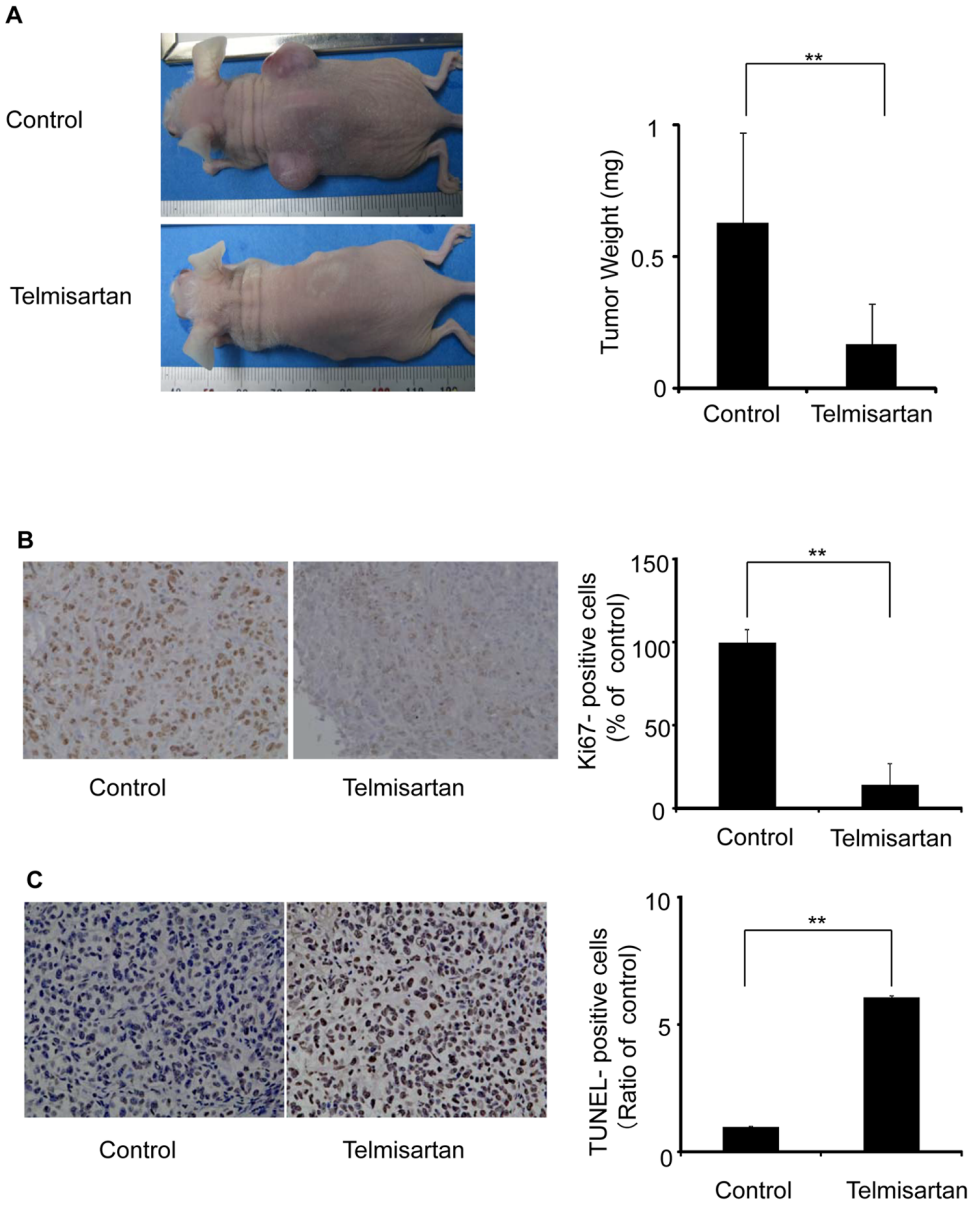
HHUA tumors in nude mice treated with telmisartan. (A) HHUA cells (5×10^6^) were bilaterally subcutaneously injected into the trunks of nude mice, forming two tumors per mouse. The mice were divided randomly into control and experimental groups. Telmisartan (100 μg/mouse) or diluent (control) was administered intraperitoneally for 5 days a week for 7 weeks. After 7 weeks of therapy, the tumors were removed from each mouse and weighed. The tumor weights in the two groups were significantly different. Columns, means; bars, SDs. **P<0.01 vs. control. (B) The effect of telmisartan on HHUA tumors in nude mice. The endometrial carcinoma cells from mice treated with telmisartan showed significantly weaker staining for Ki-67 compared to the control endometrial carcinoma cells. (C) Endometrial carcinoma cells from the mice treated with telmisartan underwent apoptosis (TUNEL-positive). All assays were performed three times. Columns, means; bars, SDs. **P<0.01 vs. control.

We tested the ability of telmisartan to inhibit the proliferation of human HHUA endometrial tumors in immunodeficient mice over the course of 7 weeks of therapy. The injection of HHUA cells (5×10^6^) led to the development of robust tumors in vivo. As shown in Figure 7A, the administration of telmisartan remarkably suppressed the growth of these tumors. All treatment groups had significantly smaller tumors compared to the diluent control groups (*P*<0.01). We measured the tumor volumes at various time points throughout treatment, and at the end of the study, the tumors were carefully dissected and weighed. The findings with regard to weight paralleled the volume measurements (data not shown). The tumor weights were 77% lower in the treatment groups than in the control cohort (*P*<0.01).

During the study, all mice were weighed and their LV pressures were recorded once weekly. Average body weights in the treatment groups were 91%–104% of the average body weight in the control group (data not shown). At baseline, the LV pressure values were not different between the groups (telmisartan group, 52.8±10.8 mmHg; control group, 52.6±11.0 mmHg) (mean ± SD). One week after administration, the mean LV pressure of the telmisartan group was significantly lower than that of the control group (50.4±7.1 mmHg vs. 68.8±13.1 mmHg, respectively; *P*<0.01). At the end of the study, the LV pressure of the control group was 68.8±6.3 mmHg and that of the telmisartan group was 58.0±3.9 mmHg (*P*<0.01).

In general, across all cohorts, all mice appeared to be healthy. No significant differences in mean weight were found between the diluent-treated mice and those that received 7 weeks of therapy (data not shown). HHUA tumors were sampled for the expression of Ki-67 by an immunohistochemical analysis, and a TUNEL assay was performed on formalin-fixed, paraffin-embedded sections. The HHUA endometrial carcinoma cells treated with telmisartan exhibited negative or focal weak staining for Ki-67 and appeared to be undergoing apoptosis (i.e., they were TUNEL-positive). The control HHUA endometrial carcinoma cells from untreated mice showed strong nuclear staining for Ki-67 and were negative for apoptosis (Fig. 7B, C).

## Discussion

Obesity, excess estrogen, type II diabetes, and hypertension are some of the important risk factors of endometrial carcinoma [4], [22]–[24]. PPARγ belongs to a family of nuclear hormone receptors that include estrogen and thyroid hormone receptors [25], [26].

The present results demonstrated that among the ARBs tested; only telmisartan was able to induce the inhibition of cell viability of endometrial cancer cell lines. The inhibitory effect of telmisartan was reduced in the presence of a PPARγ inhibitor, GW9662. It was reported there is important crosstalk between PPARγ and estrogen receptor [25], [27], [28]. Ota et al. reported that PPARγ immunoreactivity was detected in 65% of endometrial carcinoma tissue obtained from patients by immunohistochemistry. They also found that a PPARγ ligand, 15d-PGJ_2_, has antiproliferative activity in endometrial cancer cells (Ishikawa, Sawano, RL95-2 cells) [4]. Benson et al. reported that telmisartan functioned as selective PPARγ partial agonist, activating the receptor to 25% to 30% of the maximum level achieved by the full agonists pioglitazone and rosiglitazone. Other ARBs lack telmisartan’s potential for receptor interaction and have relatively little or no effect on PPARγ activity [7]. Therefore, it would be a possible mechanism, that telmisartan has anticancer effects via the PPARγ-dependent pathway.

It has also been demonstrated that telmisartan induced apoptosis and DNA fragmentation in human urological cancer [8], [9], [29]. Here we found that treatment with telmisartan significantly increased the number of apoptotic cells in all endometrial carcinoma cell lines studied. In the annexin V assay and MTP evaluation, telmisartan induced apoptosis for 48 h treatment at 100 μM. This effect was associated with a decrease in the levels of the anti-apoptotic proteins Bcl-2 and Bcl-xL. Caspase 3/7 activity also increased in response to the stimulus of 48 h telmisartan treatment at 100 μM. Our findings concerning caspase-3 cleavage and PARP cleavage suggest that this apoptosome pathway may be activated after treatment with telmisartan.

A number of cascades of signaling events are initiated in response to DSBs, to carry out apoptosis [30], [31]. Using pulse-field gel electrophoresis and immunofluorescent staining of γ-H2AX, we found that telmisaratan induced DSBs. H2AX is a member of the histone H2A family, and the phosphorylation of H2AX at serine 139, which is called “γ-H2AX,” is related to DSBs [32]–[35]. A γ-H2AX protein is considered to be the most recognizable protein in assays to measure the DNA damage [36]. A DSB is the most critical DNA damage [37], [38]. One DSB is sufficient to kill a cell, when it is not repaired [37]–[39]. In the present study, DSBs occurred in HHUA cells after 24 h of 10-μM telmisartan treatment. In contrast, Western blotting revealed that caspase 3 cleavage was not clear at 24 h of 10-μM telmisartan treatment in HHUA cells. These data suggest that DSBs could be induced before apoptosis with telmisartan treatment.

Our in vivo data indicated that apoptosis involving the tumor area occurred in the nude mice treated with telmisartan, and this antitumor activity was not accompanied by any major side effects, raising the possibility that telmisartan may serve as a useful therapy for the treatment of endometrial carcinoma. Because we started telmisartan treatment the day after malignant cells were injected into mice, we cannot conclude that telmisartan is effective against bulky tumors, which are frequently observed in the clinical setting; further studies are necessary to clarify this issue. Nonetheless, we found that telmisartan showed profound antitumor activity in vivo, and we confirmed that a small tumor burden can be eradicated by this compound in mice. This finding suggests that telmisartan therapy may be particularly effective for individuals who have minimal residual disease after curative surgery, chemotherapy, and/or radiotherapy. Although the LV pressure of the telmisartan group decreased during these experiments that of the telmisartan group was not altered for seven weeks and all mice appeared to be healthy (data not shown).

The mechanism by which telmisartan exerts its anticancer activity remains unclear. In urological cancer cells, telmisartan may mediate potent anti-proliferative effects through PPARγ [29]. In prostate cancer cells, telmisartan interacted with some signal pathways; one to block AT1R as an ARB, and the other as a transcription factor acting as the PPARγ ligand with PPARγ dependent and independent interactions [10]. As other ARBs inhibit signal transduction through epidermal growth factor (EGF) or interleukin (IL)-6 signals, telmisartan also inhibits additional signals by PPARγ activation [10], [40]. It has been reported that telmisartan inhibited the estradiol induced proliferation of ELT-3 cells (a uterine leiomyoma cell line) by acting as a PPARγ ligand, and that it inhibited angiotensin II-induced ELT-3 cell proliferation [41]. Several investigators have reported that telmisartan inhibited cell proliferation in a dose-dependent fashion in human aortic vascular smooth muscle cells [42], [43]. Yamamoto et al. reported that the antiproliferative effects of telmisartan in NIH3T3 cells lacking PPARγ were similar to those in NIH3T3 cells expressing PPARγ. They also found that telmisartan inhibited the proliferation of CHO-K1 cells, which lack AT1 receptors [42], [44] and that telmisartan can inhibit the activation of AKT in CHO-K1 cells [42]. These observations have indicated that the anticancer effect of telmisartan could be mediated, via, at least in part, PPARγ mediated pathways.

In summary, we demonstrated that telmisartan exhibited anti-proliferative activity and stimulated apoptosis in human endometrial cancer cells. The inhibition of cell proliferation in endometrial cancer cells was reduced using PPARγ antagonist, GW9662. The apoptosis events were accompanied by the down-regulation of Bcl-2, and Bcl-xL, up-regulation cleavage caspase-3, cleavage PARP followed by the induction of DSBs. In an in vivo nude mouse model, telmisartan significantly inhibited human endometrial tumor growth, without toxic side effects. It is suggested that telmisartan has anticancer effect through PPARγ dependent pathway in endometrial cancer cells. Our results showed telmisartan induced DSBs and apoptosis. Those mechanisms plays important role in anticancer effects in endometrial cancer cells. It is also suggested that telmisartan might be a new therapeutic option for the treatment of endometrial cancer.

## Supporting Information

Figure S1 **GW9662 rescue experiments with valsartan.** HHUA cells were incubated with valsartan (10 or 50 μM) for 48 h followed by preincubation with GW9662 (10 μM) for 30 min and proliferation (% of control) was measured in a WST-1 assay. Results are means ± SD of three independent experiments with triplicate dishes. GW9662 did not have any effect in HHUA cells stimulated with valsartan.
(TIF)

Figure S2 **Induction of apoptosis by telmisartan.** (A) HHUA cells were treated with 100 μM telmisartan for 48 h followed by immunostaining using an anti-γ-H2AX antibody. Control cells were treated with vehicle alone. Their nuclei were revealed by DAPI staining. The images were merged using anti-γ-H2AX antibody (red) and DAPI staining (blue). Apoptotic cells were shown by the arrow. We judged apoptotic cells by chromatin condensation, nuclear fragmentation, cellular shrinkage, apoptotic body formation with γ-H2AX positive. (B) Percentage of apoptotic cells. HHUA cells were treated with 100 μM telmisartan for 48 h. Control cells were treated with vehicle alone. The telmisartan-treated cells had significantly higher numbers of apoptotic cells compared to the untreated cells. Results = means ± SD of three independent experiments. Columns, means; bars, SDs. **P<0.01 vs. control.
(TIF)

Figure S3 **Anticancer effects via the PPARγ-dependent pathway.** The effect of the PPARγ agonist, troglitazone on the proliferation in endometrial cancer cells. HHUA endometrial cancer cell lines were treated with troglitazone at various concentrations (2.5–70 μM) or the vehicle (control) for 48 h, and proliferation (% of control) was measured in a WST-1 assay. Results are means ± SD of three independent experiments with triplicate dishes. **P<0.01 vs. control.
(TIF)

Figure S4 **The effect of telmisartan on the proliferation of human dermal fibroblast.** Human dermal fibroblast cells were treated with telmisartan at various concentrations (1–100 μM) or the vehicle (control) for 48 h, and proliferation (% of control) was measured in a WST-1 assay. Results are means ± SD of three independent experiments with triplicate dishes. **P<0.01 vs. control. Dermal fibroblast showed sensitivity to telmisartan treatment at 10 to 100 μM. However, the antiproliferative effects were significantly less than those in other cancer cell lines at 100 μM.
(TIF)

Figure S5 **Anticancer effects of telmisartan via the PPARγ-dependent pathway using some concentration of GW9662.** HHUA cells were incubated with telmisartan (10 to 100 μM) for 48 h followed by preincubation with GW9662 (10 or 50 μM) for 30 min or the vehicle (control) and proliferation (% of control) was measured in a WST-1 assay. We found that the addition of GW9662 inhibited the anticancer effects of telmisartan at 10–100 μM. Results are means ± SD of three independent experiments with triplicate dishes. *P<0.05 vs. control, **P<0.01 vs. control.
(TIF)

Figure S6 **The effect of the ARBs valsartan, losartan, candesartan and telmisartan on the proliferation of endometrial cancer cells in vitro.** HHUA cells were treated with candesartan, losartan or valsartan or telmisartan at various concentrations (1–200 μM) or the vehicle (control) for 48 h, and proliferation (% of control) was measured in a WST-1 assay. Results are means ± SD of three independent experiments with triplicate dishes. **P<0.01 vs. control. Only telmisartan significantly inhibited the cell proliferation of HHUA cells.
(TIF)
