# Supplementary figures and images for: Telmisartan Induces Growth Inhibition, DNA Double-Strand Breaks and Apoptosis in Human Endometrial Cancer Cells
Source: PLoS One. 2014 Mar 25;9(3):e93050. doi: 10.1371/journal.pone.0093050 (PMC3965508; doi:10.1371/journal.pone.0093050)

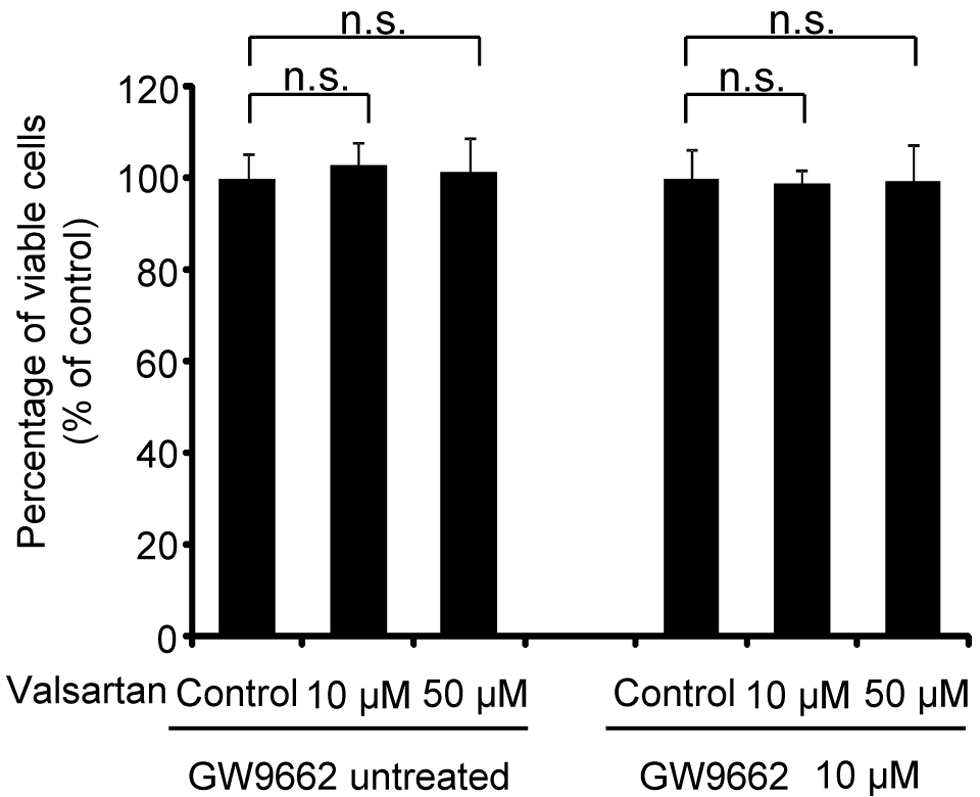

Supplement: Figure S1 — GW9662 rescue experiments with valsartan. HHUA cells were incubated with valsartan (10 or 50 μM) for 48 h followed by preincubation with GW9662 (10 μM) for 30 min and proliferation (% of control) was measured in a WST-1 assay. Results are means ± SD of three independent experiments with triplicate dishes. GW9662 did not have any effect in HHUA cells stimulated with valsartan. (TIF) [file pone.0093050.s001.tif]

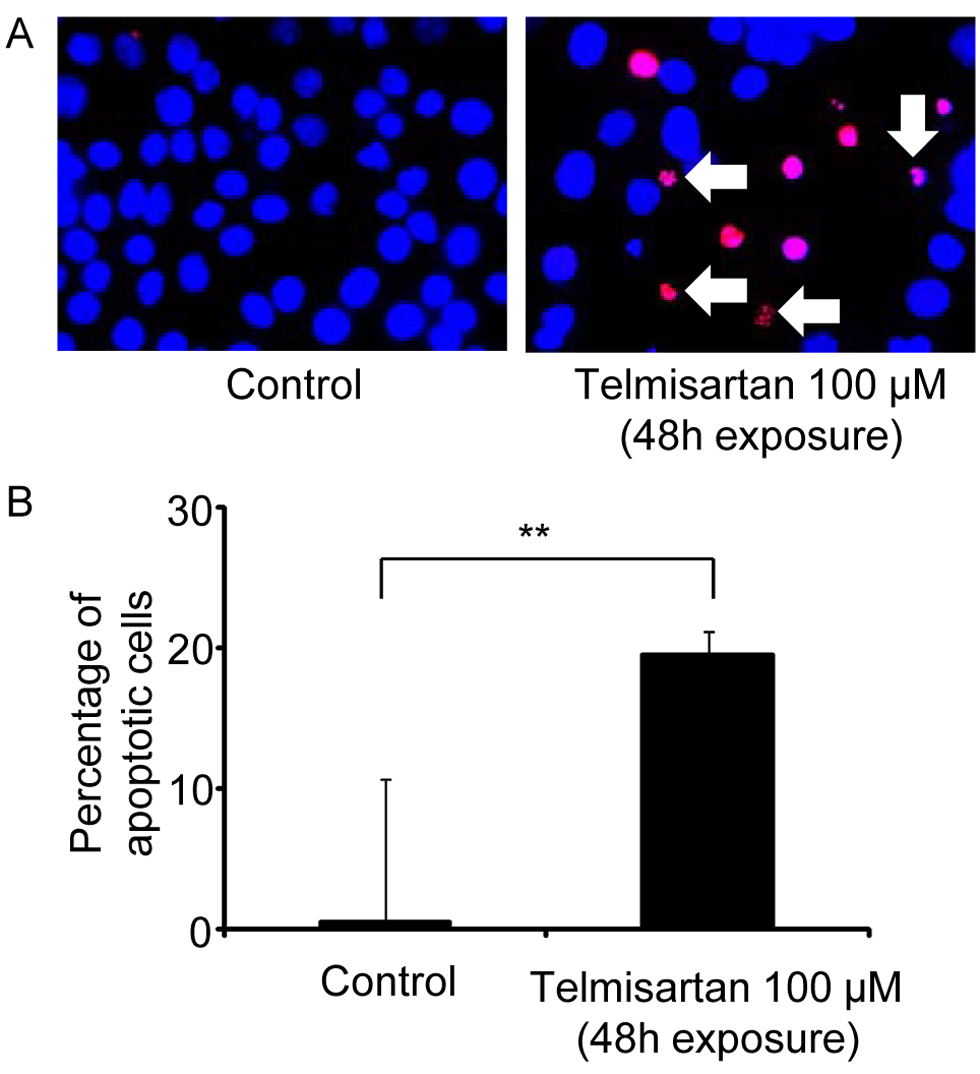

Supplement: Figure S2 — Induction of apoptosis by telmisartan. (A) HHUA cells were treated with 100 μM telmisartan for 48 h followed by immunostaining using an anti-γ-H2AX antibody. Control cells were treated with vehicle alone. Their nuclei were revealed by DAPI staining. The images were merged using anti-γ-H2AX antibody (red) and DAPI staining (blue). Apoptotic cells were shown by the arrow. We judged apoptotic cells by chromatin condensation, nuclear fragmentation, cellular shrinkage, apoptotic body formation with γ-H2AX positive. (B) Percentage of apoptotic cells. HHUA cells were treated with 100 μM telmisartan for 48 h. Control cells were treated with vehicle alone. The telmisartan-treated cells had significantly higher numbers of apoptotic cells compared to the untreated cells. Results = means ± SD of three independent experiments. Columns, means; bars, SDs. **P<0.01 vs. control. (TIF) [file pone.0093050.s002.tif]

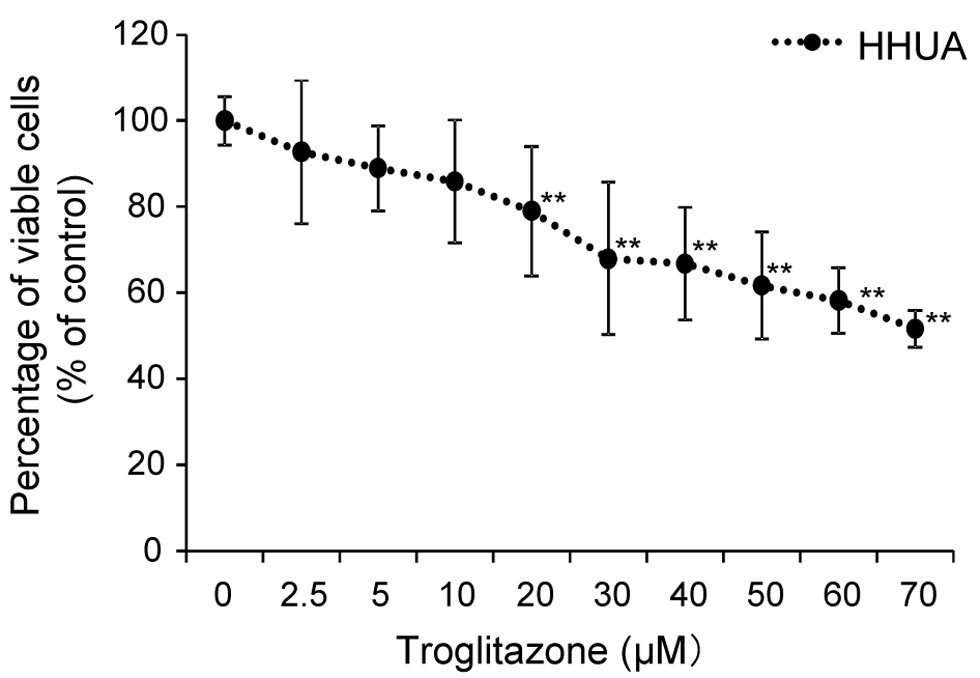

Supplement: Figure S3 — Anticancer effects via the PPARγ-dependent pathway. The effect of the PPARγ agonist, troglitazone on the proliferation in endometrial cancer cells. HHUA endometrial cancer cell lines were treated with troglitazone at various concentrations (2.5–70 μM) or the vehicle (control) for 48 h, and proliferation (% of control) was measured in a WST-1 assay. Results are means ± SD of three independent experiments with triplicate dishes. **P<0.01 vs. control. (TIF) [file pone.0093050.s003.tif]

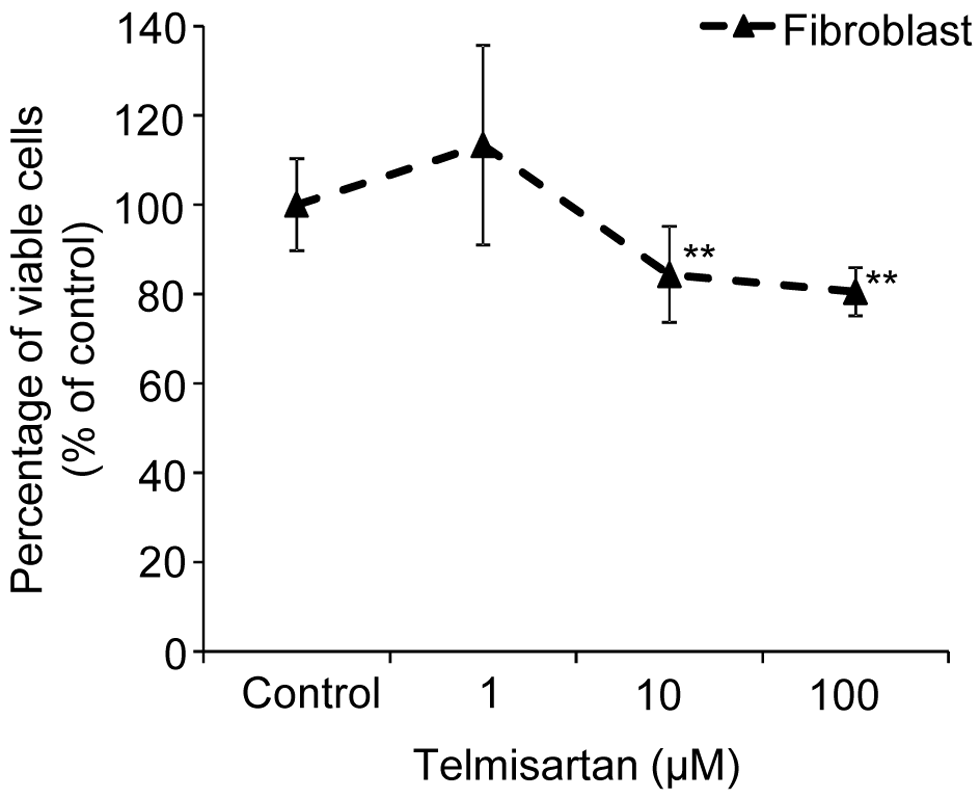

Supplement: Figure S4 — The effect of telmisartan on the proliferation of human dermal fibroblast. Human dermal fibroblast cells were treated with telmisartan at various concentrations (1–100 μM) or the vehicle (control) for 48 h, and proliferation (% of control) was measured in a WST-1 assay. Results are means ± SD of three independent experiments with triplicate dishes. **P<0.01 vs. control. Dermal fibroblast showed sensitivity to telmisartan treatment at 10 to 100 μM. However, the antiproliferative effects were significantly less than those in other cancer cell lines at 100 μM. (TIF) [file pone.0093050.s004.tif]

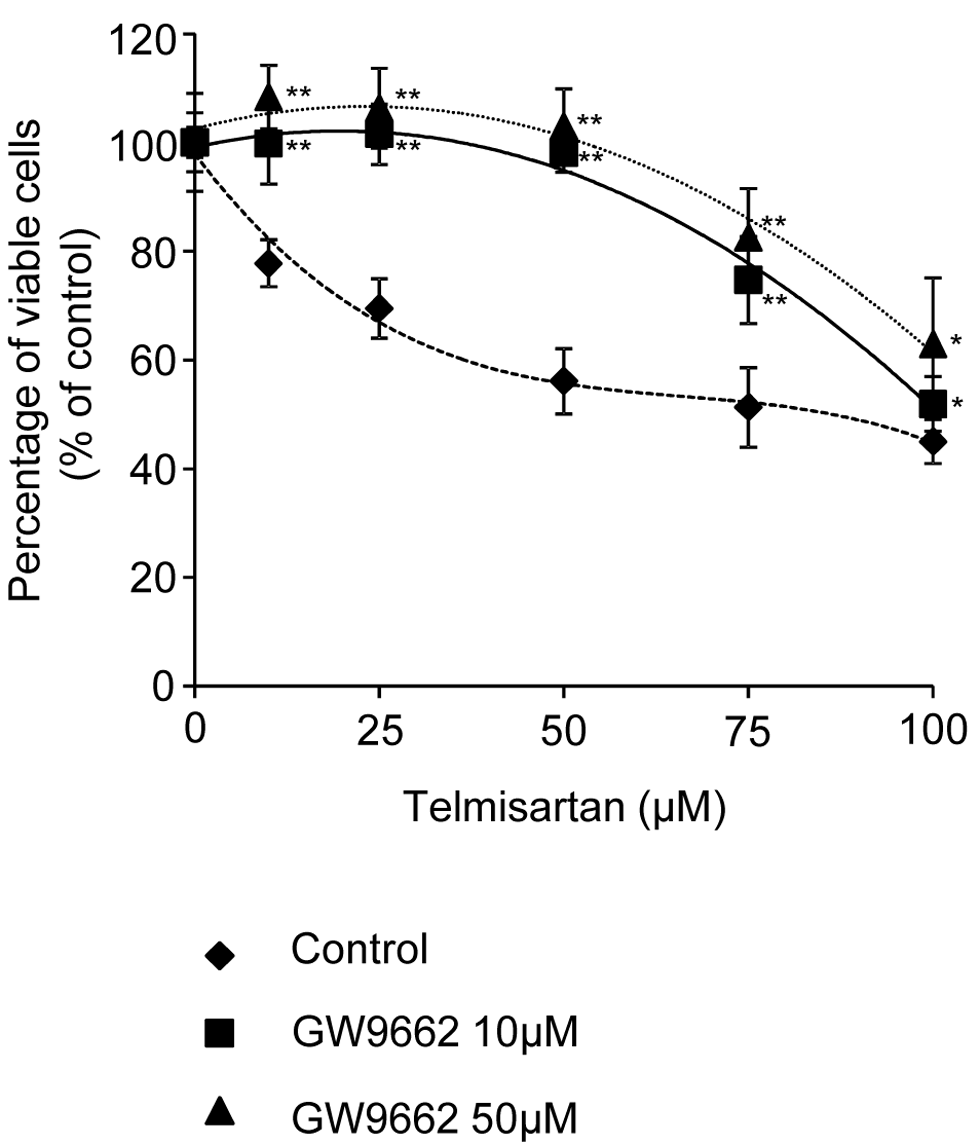

Supplement: Figure S5 — Anticancer effects of telmisartan via the PPARγ-dependent pathway using some concentration of GW9662. HHUA cells were incubated with telmisartan (10 to 100 μM) for 48 h followed by preincubation with GW9662 (10 or 50 μM) for 30 min or the vehicle (control) and proliferation (% of control) was measured in a WST-1 assay. We found that the addition of GW9662 inhibited the anticancer effects of telmisartan at 10–100 μM. Results are means ± SD of three independent experiments with triplicate dishes. *P<0.05 vs. control, **P<0.01 vs. control. (TIF) [file pone.0093050.s005.tif]

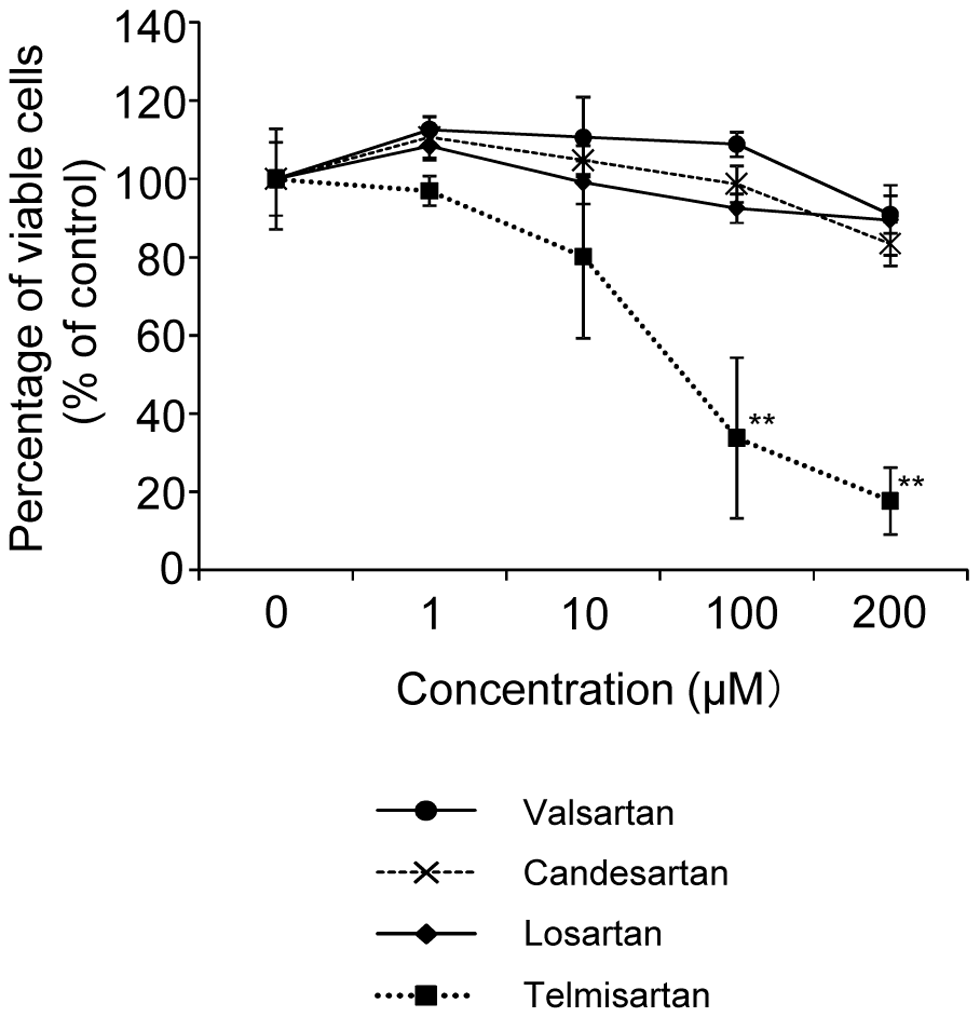

Supplement: Figure S6 — The effect of the ARBs valsartan, losartan, candesartan and telmisartan on the proliferation of endometrial cancer cells in vitro. HHUA cells were treated with candesartan, losartan or valsartan or telmisartan at various concentrations (1–200 μM) or the vehicle (control) for 48 h, and proliferation (% of control) was measured in a WST-1 assay. Results are means ± SD of three independent experiments with triplicate dishes. **P<0.01 vs. control. Only telmisartan significantly inhibited the cell proliferation of HHUA cells. (TIF) [file pone.0093050.s006.tif]
